# Personality traits are associated with the development and severity of chemosensory alterations during chemotherapy: a longitudinal study

**DOI:** 10.1007/s00520-026-11074-2

**Published:** 2026-08-10

**Authors:** Angelica Lippi, Agnès Giboreau, Véronique Mourier, Christophe Hebert, Renaud Schiappa, Amélie Cougny, Kouceila Touati, Colin Debaigt, Anasthasia Gama, Francesco Claudio Stingo, Mathieu Piquet, Erminio Monteleone, Sara Spinelli

**Affiliations:** 1 SensoryLab, DAGRI – Department of Agriculture, Food, Environment and Forestry, University of Florence, Via G. Donizetti, 6, 50144 Florence, Italy; 2 Institut Lyfe Research & Innovation Center, 1 A Chemin de Calabert, 69130 Ecully, France; 3 Health, Systemic, Process, UR4129 Unit Research, University of Lyon, University Claude Bernard Lyon 1, 69008 Lyon, France; 4 Elior Santé, 51 Chemin Des Mèches, 94000 Créteil, France; 5 Department of Medical Oncology, Centre Antoine Lacassagne - Université Côte d’Azur, Nice, France; 6 Department of Epidemiology, Biostatistics and Health Data (DEBDS), Centre Antoine Lacassagne - Université Côte d’Azur, Nice, France; 7 Department of Clinical Research and Innovation (DRCI), Centre Antoine Lacassagne - Université Côte d’Azur, Nice, France; 8 Department of Statistics, Computer Science, Applications “Giuseppe Parenti” (DiSIA), University of Florence, Viale Morgagni, 59, 50134 Florence, Italy

**Keywords:** Cancer, Chemotherapy, Sensory alterations, Personality traits, Food neophobia

## Abstract

**Purpose:**

Chemotherapy-induced sensory alterations are common and may negatively affect nutritional status, treatment adherence and quality of life in people with cancer. However, inter-individual variability in these alterations remains poorly understood. This study examined whether personality traits, food neophobia, disgust sensitivity, and sensitivity to reward, contribute to variability in self-reported sensory alterations during chemotherapy.

**Methods:**

A longitudinal study was conducted in 95 patients undergoing chemotherapy, assessed at baseline (T0) and after nine weeks of treatment (T1). Sensory alterations were measured using a modified version of the Chemotherapy-Induced Taste Alteration Scale, and personality traits were assessed with validated questionnaires. Changes over time were evaluated using repeated-measures analyses, while associations between predictors and outcomes were examined using ANCOVA and multivariable logistic regression models.

**Results:**

After nine weeks, the prevalence of self-reported sensory alterations increased across all domains (24–60%). Discomfort, nausea, phantogeusia/parageusia and general taste alterations were the most frequently reported symptoms. Personality traits were significantly associated with several outcomes. Food neophobia was positively associated with the likelihood of reporting general taste alterations. In contrast, sensitivity to reward was consistently inversely associated with phantogeusia/parageusia and nausea.

**Conclusions:**

Personality traits are associated with individual differences in chemotherapy-induced chemosensory alterations. Identifying at-risk patients may support targeted nutritional and behavioural interventions to improve treatment tolerance and well-being.

**Supplementary Information:**

The online version contains supplementary material available at https://doi.org/10.1007/s00520-026-11074-2.

## Introduction

Chemotherapy is a well-established cause of sensory alterations, which can negatively affect nutritional status, food enjoyment, treatment adherence, and quality of life in people with cancer [1, 2]. These alterations are highly prevalent, affecting 30% to 70% of patients depending on cancer type, treatment modality, and assessment methods [3, 4]. Taste and smell changes often emerge early during treatment and tend to intensify over time, with important consequences for dietary preferences and emotional responses to food [5, 6]. Taste alterations may include both quantitative (e.g., hypergeusia, hypogeusia) and qualitative disturbances (e.g., dysgeusia, phantogeusia, parageusia), while olfactory changes can similarly involve both reduced sensitivity and distortions [7–10]. These alterations are commonly assessed through self-reported measures and can substantially disrupt patients’ daily lives [11–13].

Despite their clinical relevance, the predictors of chemosensory alterations remain incompletely understood. Previous research has identified potential clinical and demographic factors, such as chemotherapy type, treatment duration, oral health status, sex, and age, but findings are inconsistent across studies [7, 11, 12, 14–19]. This variability suggests that additional individual factors may contribute to differences in sensory experiences.

Personality, defined as relatively stable patterns of emotional, cognitive, and behavioural functioning [20], may represent an important but underexplored source of inter-individual variability. Evidence from healthy populations indicates that personality traits influence food perception, evaluation, and acceptance [21–23] suggesting a potential role also in clinical contexts.

In the present study, three personality traits were considered: food neophobia, disgust sensitivity, and sensitivity to reward. Food neophobia, defined as reluctance to try unfamiliar foods [24], is associated with heightened vigilance and avoidance toward food-related stimuli, as well as increased sensitivity to warning sensations such as bitterness or astringency [25–30]. Disgust sensitivity reflects individual differences in aversive reactivity to potentially harmful stimuli and has been linked to stronger rejection of unpleasant tastes, textures, and food-related contaminants, as well as enhanced interoceptive awareness [29–33]. In contrast, sensitivity to reward, conceptualized within Gray’s Reinforcement Sensitivity Theory, is associated with approach behaviour and responsiveness to rewarding stimuli [34, 35]. In food contexts, it is linked to stronger hedonic responses and greater motivation to engage with food, which may attenuate the perceived impact of sensory alterations [36, 37]. Taken together, these traits capture complementary dimensions of food-related experience, avoidance (food neophobia), aversive reactivity (disgust sensitivity), and approach motivation (reward sensitivity), that may shape how individuals perceive and report chemosensory alterations. To the best of our knowledge, no study has previously examined whether these personality traits are associated with chemosensory alterations in cancer patients undergoing chemotherapy.

The present study aimed to investigate whether food neophobia, disgust sensitivity, and sensitivity to reward contribute to individual differences in the worsening and severity of self-reported chemosensory alterations in people with cancer undergoing chemotherapy.

It was hypothesized that higher food neophobia and disgust sensitivity would be associated with increased likelihood and severity of alterations, whereas higher sensitivity to reward would be associated with reduced likelihood and severity.

## Materials and methods

### Study design

This longitudinal study included two assessment time points: baseline (T0; prior to chemotherapy initiation), and after nine weeks of treatment (T1; approximately three chemotherapy cycles), a time frame in which sensory alterations are expected to peak [38]. All participants were assessed within a narrow timeframe after diagnosis (approximately one week), minimizing variability in time since diagnosis.

At T0, demographic data, personality traits, and self-reported sensory alterations were collected. At T1, sensory alterations were reassessed. The study was approved by the Comité de Protection des Personnes Nord Ouest IV (No. 23.00862.000179; ID RCB 2023-A00259-36; registered on August 21, 2023, ClinicalTrials.gov; NCT06058312) and conducted in accordance with the Declaration of Helsinki. All participants provided written informed consent.

### Participants

Participants were recruited at the Centre Antoine Lacassagne Hospital (Nice, France) between 2023 and 2024. Eligibility criteria included: diagnosis of cancer, age 18–70 years, scheduled to begin chemotherapy, and no chemotherapy in the previous three months. Exclusion criteria included inability to eat, enteral/parenteral nutrition, prior gastric surgery, head/neck radiotherapy, upper aerodigestive tract cancers, and insufficient French proficiency. Of the 110 patients enrolled, 95 completed the study (mean age = 53 years, SD = 10.17; 86% female) (Table 1). The predominance of female participants reflects the case mix of the recruitment centre, where breast and gynaecological cancers account for most patients receiving chemotherapy.

**Table 1:** Demographic and clinical data of the participants

		**Males**	**Females**	**Total**
**Sex**		13 (14%)	82 (86%)	95 (100%)
**Age (years)**				
21—30		1 (8%)	3 (4%)	4 (4%)
31—40		0 (0%)	8 (10%)	8 (9%)
41—50		1 (8%)	19 (23%)	20 (21%)
51—60		7 (54%)	29 (35%)	36 (38%)
61—70		4 (30%)	23 (28%)	27 (28%)
**Cancer type**				
Breast		0 (0%)	57 (69.5%)	57 (60%)
Gynaecological		0 (0%)	14 (17.1%)	14 (15%)
Gastrointestinal		7 (53.8%)	5 (6.1%)	12 (13%)
Respiratory		4 (30.8%)	5 (6.1%)	9 (9%)
Genitourinary (male)		1 (7.7%)	0 (0%)	1 (1%)
Other/Mixed		1 (7.7%)	1 (1.2%)	2 (2%)
**Chemotherapy regimen**	**Chemotherapy type**			
Platinum-based	platinum-taxane, platinum-antimetabolite, platinum	10 (10.5%)	33 (34.7%)	43 (45.3%)
Anthracycline-based	anthracycline, anthracycline-alchilant	0 (0%)	35 (36.8%)	35 (36.8%)
Taxane-based	taxane, taxane alchilant	2 (2.1%)	11 (11.6%)	13 (13.7%)
Other (target)	deruxtecan, antimetabolite	1 (1.1%)	3 (3.2%)	4 (4.2%)

### Measures

#### Personality traits

To operationalize the psychological constructs introduced in the theoretical framework, validated psychometric scales were used to assess food neophobia, disgust sensitivity, and sensitivity to reward (Table 2). These constructs represent relatively stable personality traits rather than transient emotional states. These variables were included as key predictors of individual differences in the perception and reporting of chemosensory alterations.

**Table 2:** Definitions and clinical implications of personality traits

	**Definition**	**Potential clinical implications**
**Food neophobia**	A stable tendency to avoid or hesitate to try unfamiliar foods. People with high food neophobia generally prefer familiar foods and are less willing to experiment with new tastes or dietary changes	Food neophobia is consistently associated with lower dietary variety, reduced acceptance of novel foods, and lower consumption of fruits and vegetables In patients undergoing chemotherapy, this trait may reduce willingness to try alternative foods or dietary strategies when familiar foods become unpalatable because of treatment-related taste and smell changes, potentially compromising dietary adequacy and nutritional management
**Disgust sensitivity**	A stable individual characteristic in how strongly a person experiences feelings of disgust when exposed to stimuli perceived as unpleasant, aversive, or potentially contaminating	Individuals with higher disgust sensitivity may be more likely to reject certain foods because of their taste, smell, texture, appearance, or associations with contamination or illness In clinical settings, this may influence food acceptance, dietary variety, and responses to treatment-related changes in sensory perception or gastrointestinal symptoms
**Sensitivity to reward**	A stable personality trait reflecting how strongly a person is motivated by rewarding or pleasurable experiences, including eating. Individuals with higher sensitivity to reward generally experience stronger motivation to seek and respond to rewarding food-related cues	Higher sensitivity to reward has been associated with stronger responsiveness to food cues, greater appetitive motivation, and better positive psychological functioning In patients receiving chemotherapy, this trait may help maintain engagement with eating despite sensory changes, potentially reducing the perceived impact of treatment-related chemosensory alterations

Food neophobia was assessed using the 10-item Food Neophobia Scale previously translated and used in French populations [24, 26], with responses on a 7-point Likert scale (1 = strongly disagree; 7 = strongly agree; e.g. “I am constantly sampling new and different foods”); total scores, calculated as sum, range from 10 to 70. Higher scores indicate stronger food neophobia, reflecting greater avoidance and vigilance toward unfamiliar food-related stimuli.

Disgust sensitivity was measured using the 8-item Disgust Sensitivity Scale–Short Form [32, 39]. translated into French using a forward–backward procedure. Items were rated either on a 5-point Likert scale (1 = strongly disagree; 5 = strongly agree; e.g. “I might be willing to try eating monkey meat under some circumstances”) or on a 5-point disgust scale (1 = not at all disgusting; 5 = extremely disgusting), with total scores, calculated as sum, ranging from 8 to 40, reflecting individual differences in aversive reactivity to potentially harmful or unpleasant sensory stimuli.

Sensitivity to reward was assessed using the reward subscale of the French short version of the Sensitivity to Punishment and Sensitivity to Reward Questionnaire [40, 41] consisting of 17 items rated on a 4-point scale (1 = totally no; 4 = totally yes; e.g. “Do you often do things to be praised?”). Total scores were calculated as sum (recoded as 0 = 1/2 and 1 = 3/4 on the scale), ranging from 1 to 17, reflecting individual differences in approach motivation and responsiveness to rewarding stimuli.

#### Chemosensory alterations

Self-reported sensory alterations were assessed using a modified version of the French version of the Chemotherapy-induced Taste Alterations Scale – modified, including additional items and altered structure [42]. The scale was adapted with added items on olfactory difficulties (as proposed by Drareni et al., 2021 [2]), hypergeusia and metallic taste (based on preliminary qualitative studies). The item on umami was removed due to low familiarity among participants with this concept. The final version included 24 items, each rated on a 5-point scale (1 = not at all, 5 = very much; e.g. “Have a bad taste in the mouth”), grouped into eight subscales representing distinct dimensions of chemotherapy-related chemosensory alterations: sweet/salty decline (reduced perception of sweet and salty tastes), sour/bitter decline (reduced perception of sour and bitter tastes), hypergeusia (increased taste intensity), odour decline (reduced smell perception), general taste alterations (overall disturbances in taste perception), phantogeusia/parageusia (respectively, phantom or distorted taste sensations), nausea (treatment-related nausea affecting eating), and discomfort (food-related discomfort during eating). Subscale scores were calculated as the mean of item responses, with values above 1 indicating a perceived alteration and higher scores reflecting greater severity. Details about the scale used are reported in the Supplementary Materials.

### Data analysis

Descriptive analyses were conducted for all study variables. Internal consistency of all questionnaires was assessed using Cronbach’s α to ensure reliability. To further evaluate the factor structure and validity of the French versions of the Food Neophobia Scale, Disgust Sensitivity Scale–Short Form and the subscale of Sensitivity to Reward of the Sensitivity to Punishment and Sensitivity to Reward Questionnaire and the modified Chemotherapy-induced Taste Alterations Scale, exploratory factor analysis was employed. Promax rotation was used for the modified Chemotherapy-induced Taste Alterations Scale. The Kaiser–Meyer–Olkin (KMO) and Bartlett’s test of sphericity test was conducted to assess the suitability of the data for factor analysis. Pearson correlations were used for the Food Neophobia Scale and Disgust Sensitivity Scale–Short Form scales, while polychoric correlations were applied for the Sensitivity to Punishment and Sensitivity to Reward Questionnaire, given the nature of the data. A threshold value of 0.28 was used in factor analysis to retain an item within a factor.

Different statistical models addressed complementary research questions. Repeated-measures ANOVA was used to evaluate longitudinal changes in chemosensory alterations, baseline-adjusted ANCOVA to identify predictors of alteration severity at T1 while controlling for baseline values, and binary logistic regression to identify predictors of worsening chemosensory alterations.

In particular, repeated-measures ANOVA models with Satterwhaite correction (REML estimation method, compound symmetry) were conducted to evaluate changes in self-reported chemosensory alterations between baseline (T0) and 9 weeks after chemotherapy initiation (T1), with age and sex as covariate on the different types of alterations measured with the modified Chemotherapy-induced Taste Alterations Scale (established significance was p < 0.05).

To identify factors associated with the severity of chemosensory alterations at T1, baseline-adjusted ANCOVA models were performed with T1 modified Chemotherapy-induced Taste Alterations Scale subscale scores as dependent variables. Personality traits (food neophobia, core visceral disgust sensitivity, and sensitivity to reward), age, sex, and treatment type were included as independent variables, while the corresponding baseline (T0) subscale score was entered as a covariate. Type III sums of squares were used to test the significance of each predictor after adjustment for all other variables included in the model. Multicollinearity among predictors was assessed using variance inflation factors (VIF) and tolerance values.

Finally, binary logistic regression analyses were performed to examine whether age, sex, treatment type, and personality traits were associated with the likelihood of experiencing worsening self-reported chemosensory alterations (sweet/salty decline, sour/bitter decline, hypergeusia, odour decline, general taste alterations, phantogeusia/parageusia, nausea and discomfort) at T1. For each modified Chemotherapy-induced Taste Alterations Scale subscale, participants were classified as having experienced worsening if their change score (T1 − T0) was greater than 0, without considering the severity of the reported alteration. Odds ratios (ORs) and 95% confidence intervals (95% CIs) were calculated for all predictors. Model fit was assessed using the Hosmer–Lemeshow test, while model performance was evaluated through sensitivity, specificity, and the area under the receiver operating characteristic (ROC) curve (AUC). Given the relatively limited sample size and the number of outcome events available for some logistic regression models, these analyses were considered exploratory and were primarily intended to identify potential demographic, clinical, and personality factors associated with worsening self-reported chemosensory alterations. No formal adjustment for multiple comparisons was applied because the analyses were hypothesis-driven and each statistical model addressed a distinct research question rather than repeated testing of the same hypothesis. Therefore, the results should be interpreted in the context of the overall pattern of findings rather than based on individual *p*-values alone.

## Results

### Psychometrics of the scales

The modified Chemotherapy-induced Taste Alterations Scale data were suitable for factor analysis (KMO = 0.84; Bartlett’s test χ^2^ (276) = 1825.88, *p* < 0.001). After Promax rotation, the questionnaire showed a structure broadly consistent with the original version [42], with some differences. Items loaded on five factors: (1) general taste alterations and sweet/salty decline; (2) odour items; (3) bitter/sour decline, including weak loading of the hypergeusia item; (4) discomfort items (excluding nausea); and (5) nausea together with phantogeusia/parageusia. Based on these results, nausea was considered separately from discomfort, and hypergeusia and taste declines were treated as distinct constructs (see Supplementary Materials). Cronbach’s α values were above 0.8 for all factors.

The Food Neophobia Scale showed good internal reliability (α = 0.83), while Disgust Sensitivity Scale–Short Form (α = 0.74) and Sensitivity to Reward Questionnaire (α = 0.75) showed acceptable reliability, consistent with previous findings [36].

### Worsening and severity of self-reported alterations after nine weeks of chemotherapy

**Fig. 1:**
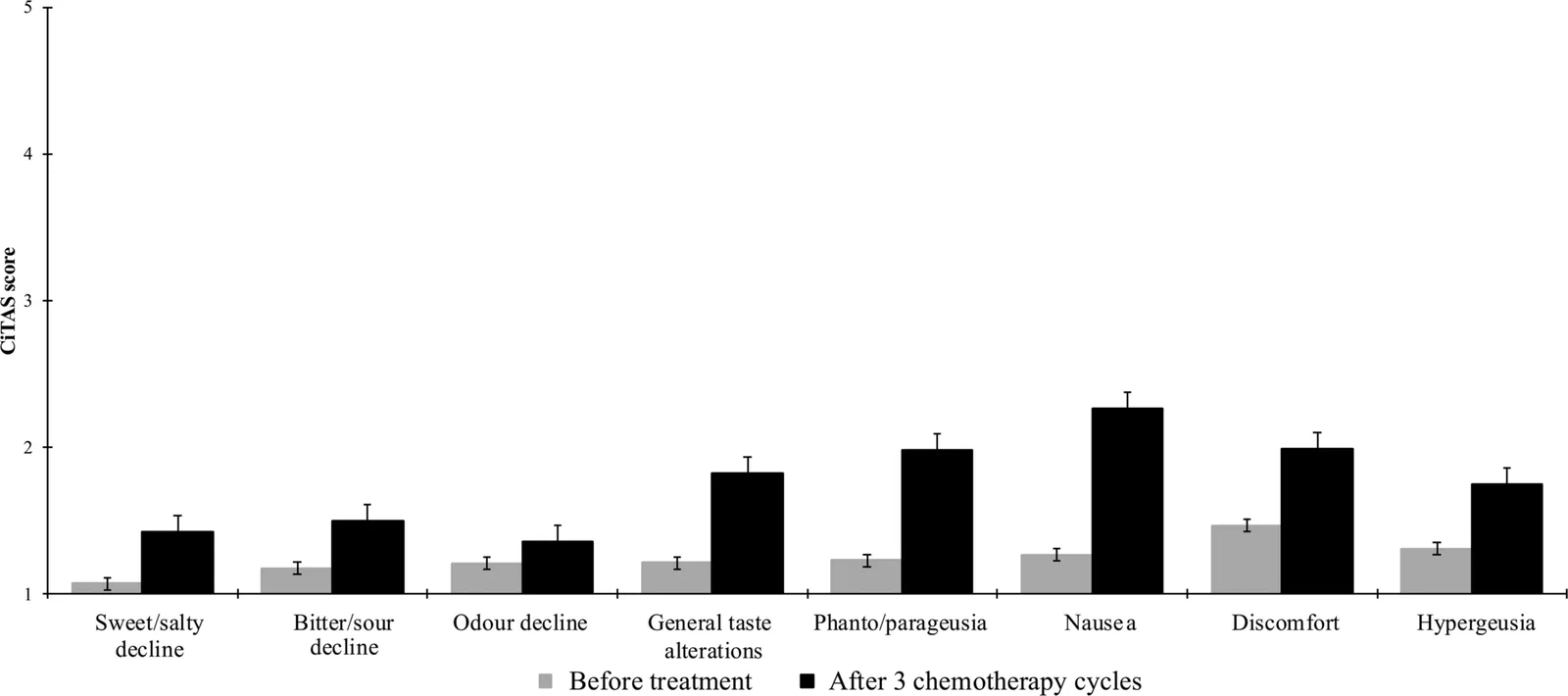
Modified Chemotherapy-induced Taste Alterations Scale scores mean ratings for alteration type at T0 (before treatment) and T1 (after 9 weeks since the beginning of the treatment/after 3 chemotherapy cycles). Higher scores indicate higher level of alteration of the modified Chemotherapy-induced Taste Alterations Scale (1 = not at all; 5 = very much).

Treatment had a significant effect on all modified Chemotherapy-induced Taste Alterations Scale subscales (Fig. 1 and Table 2 s, Supplementary Materials). After nine weeks, patients reported higher levels of sweet/salty decline (F _(1,96)_ = 19.01; *p* < 0.0001), bitter/sour decline (F _(1,96)_ = 10.52; *p* = 0.002), odour decline (F _(1,96)_ = 4.14; *p* = 0.045), general taste alterations (F _(1,96)_ = 30.69; *p* < 0.0001), phantogeusia/parageusia (F _(1,96)_ = 33.54; *p* < 0.0001), nausea (F _(1,96)_ = 37.86; *p* < 0.0001), discomfort (F _(1,96)_ = 30.34; *p* < 0.0001) and hypergeusia (F _(1,96)_ = 9.51; *p* = 0.003). Age was positively associated with odour decline (t = 1.98, F _(1,96)_ = 3.94; *p* = 0.05), and with general taste alterations (t = 2.03, F _(1,96)_ = 4.11; *p* = 0.045), while sex and chemotherapy regimen showed no significant effects. Although treatment significantly affected mean responses, the repeated covariance structure did not improve model fit, except for discomfort (*p* < 0.0001) and marginally for hypergeusia (*p* = 0.055) and phantogeusia/parageusia (*p* = 0.083). Mean shifts are shown in Fig. 1.

The most frequent increases from baseline after nine weeks were observed for discomfort (60.0%) and nausea (55.8%), followed by phantogeusia/parageusia (51.6%) and general taste alterations (45.3%). Lower worsening rates were found for bitter/sour decline (32.6%), sweet/salty decline (26.3%), odour decline (24.2%), and hypergeusia (26.3%).

### Factors affecting prevalence, worsening, and severity of chemosensory alterations

Correlations among predictors were low (maximum r = 0.33, *p* = 0.001 between food neophobia and disgust sensitivity), Multicollinearity diagnostics indicated no evidence of problematic collinearity among the predictors (all VIFs < 1.5; all tolerance values > 0.70). Although food neophobia and disgust sensitivity are related, they are distinct constructs, as food neophobia also involves responses to food novelty and perceived risk beyond high negative emotions [43].

Baseline-adjusted ANCOVA models indicated that higher sensitivity to reward was significantly associated with lower severity of general taste alterations (B = −0.09, t = −2.57, *p* = 0.012, partial η^2^ = 0.072) and phantogeusia/parageusia (B = −0.10, t = −2.24, *p* = 0.028, partial η^2^ = 0.056). There was also a trend towards an association between higher disgust sensitivity and greater phantogeusia/parageusia severity, although this did not reach statistical significance (B = 0.042, t = 1.79, *p* = 0.077). The overall ANCOVA model for nausea did not reach statistical significance (F _(9,85)_ = 1.26, *p* = 0.273). However, after adjustment for baseline nausea, age, sex, and treatment, higher sensitivity to reward remained independently associated with lower nausea severity at T1 (F _(1,85)_ = 5.30, *p* = 0.024, B = −0.11, partial η^2^ = 0.059). Furthermore, odour decline at T1 was significantly associated with baseline odour decline (B = 0.514, t = 4.70, *p* < 0.0001, partial η^2^ = 0.206), older age (B = 0.015, t = 2.16, *p* = 0.034, partial η^2^ = 0.052), and therapy type (F_(3,85)_ = 2.81, *p* = 0.044, partial η^2^ = 0.090). Compared with taxane-based therapy (reference category), anthracycline-based (B = 0.625, t = 2.66, *p* = 0.009) and platinum-based therapies (B = 0.506, t = 2.16, *p* = 0.034) were associated with greater odour decline.

**Fig. 2:**
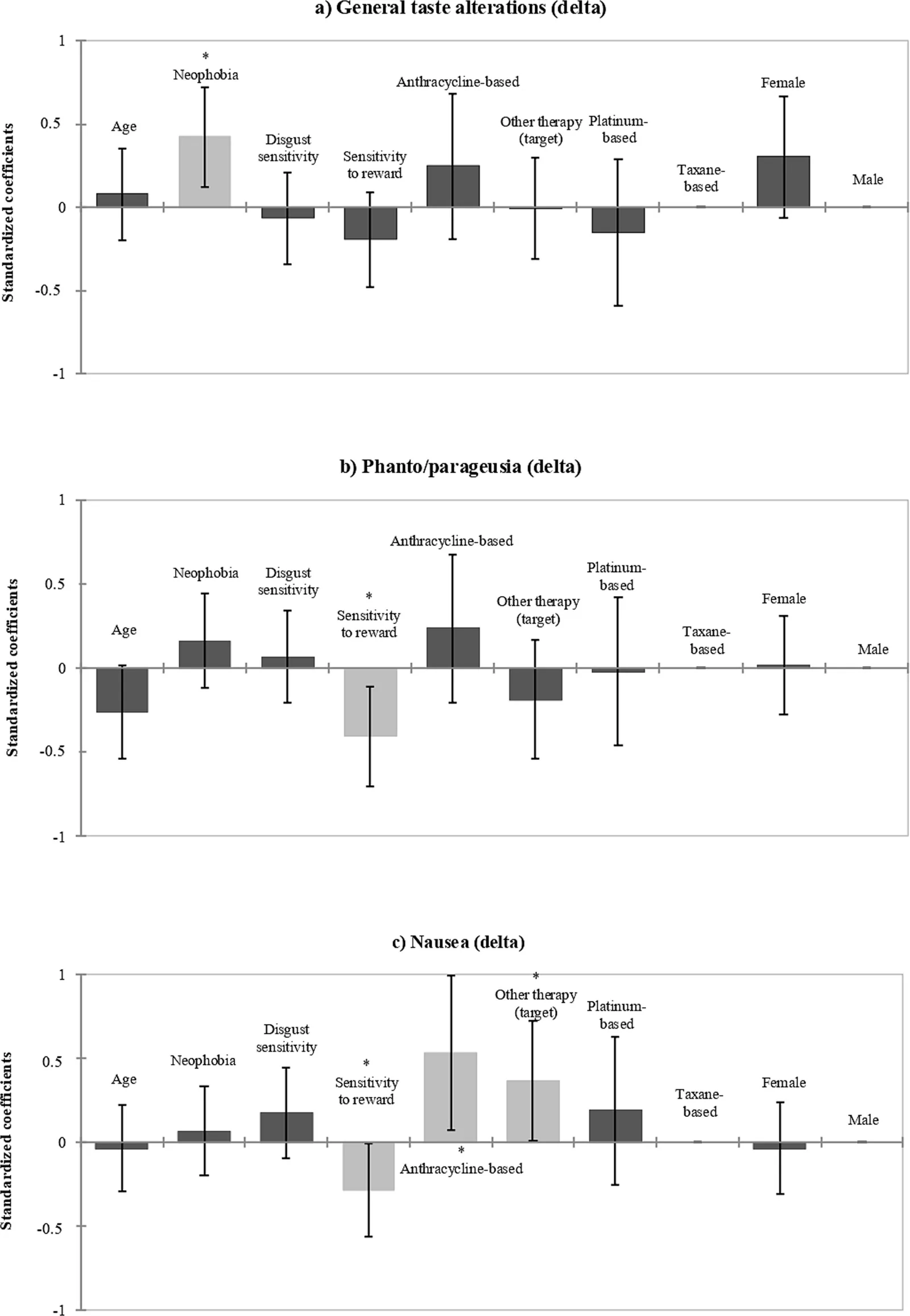
Standardized regression coefficients (95% conf. interval) from logistic regression of the group with/without alterations in (**a**) general taste alterations, **b**) phanto/parageusia and **c**) nausea explained by personality traits, age, sex and chemotherapy type: Model was computed coding Yes alteration = 1 and No alteration = 0, negative coefficients indicate lower odds of reporting an alteration; positive coefficients indicate higher odds. * Indicates *p* ≤ 0.05

Binary logistic regression analyses showed that psychological traits, particularly food neophobia and sensitivity to reward, were the most consistent predictors of the worsening, whereas demographic variables had limited influence (Fig. 2). A significant model was found for general taste alterations (χ^2^ (8) = 21.76, *p* = 0.005; Nagelkerke R^2^ = 26.2%), with food neophobia as a positive predictor (OR = 1.07, 95% CI [1.02, 1.13], *p* = 0.006), indicating that each unit increase in neophobia was associated with a 7% increase in the odds of reporting general taste alterations (Fig. 2a).

For phantogeusia/parageusia, the model was also significant (χ^2^ (8) = 22.09, *p* = 0.005), with sensitivity to reward emerging as a significant negative predictor (OR = 0.79, 95% CI [0.66, 0.94], *p* = 0.008), indicating that each unit increase in sensitivity to reward was associated with a 21% decrease in the odds of reporting the outcome and thus suggesting an association (Fig. 2b). Similarly, in the model predicting nausea (χ^2^ (8) = 16.80, *p* = 0.032), sensitivity to reward was negatively associated with the outcome (OR = 0.84, 95% CI [0.72, 1.00], *p* = 0.045) indicating that higher reward sensitivity was associated with a modest reduction in the odds of the outcome, while therapy type increased odds for anthracycline-based (OR = 7.07, *p* = 0.024) and targeted therapies (OR = 15.17, *p* = 0.047), although estimates were imprecise (Fig. 2c).

Sensitivity to reward also predicted lower odds of reporting hypergeusia (OR = 0.81, *p* = 0.034). No significant predictors were identified for bitter/sour decline, odour decline, discomfort, or sweet/salty decline. Age showed a borderline positive association with odour decline and a negative association with phanto/parageusia (*p* < 0.1), while sex showed a borderline effect on discomfort (OR = 4.60, *p* = 0.056), with wide confidence intervals.

Model discrimination ranged from moderate to good (AUC = 0.70–0.77). The model for general taste alterations showed balanced performance (accuracy = 65.3%, sensitivity = 60.5%, specificity = 69.2%), while phantogeusia/parageusia showed higher sensitivity (75.5%). The nausea model showed high sensitivity (79.3%) but lower specificity (54.8%). The hypergeusia model showed high accuracy (74.7%) but poor sensitivity (12.0%), likely due to class imbalance. A summary of the effects of personality traits on chemosensory alteration is provided in Table 3.

**Table 3:** Summary table of key associations (personality trait, CiTAS subscale, analysis type, direction of effect, and significance level)

**Personality trait**	**Associated CiTAS subscale(s)**	**Analysis type**	**Direction of effect**	**Significance level**
**Food Neophobia**	General taste alterations	Logistic regression	Positive association: higher food neophobia predicted increased odds of developing general taste alterations	*p* = 0.006
**Sensitivity to Reward**	General taste alterations, phantogeusia/parageusia, nausea	Baseline-adjusted ANCOVA, logistic regression	Negative association: higher reward sensitivity predicted lower severity, and lower odds of developing several chemosensory alterations	*p* = 0.005—0.034 partial η^2^ = 0.056—0.072

Only statistically significant associations (*p* < 0.05) are reported

## Discussion

This study investigated whether individual factors such as personality traits (food neophobia, disgust sensitivity and sensitivity to reward ) help explain why some patients report taste and smell changes more strongly than others, after controlling for chemotherapy regimen, sex and age. Our findings demonstrate that chemotherapy significantly increased chemosensory alterations after nine weeks of treatment, consistent with previous findings indicating that side effects peak during the third cycle [38]. The study hypotheses were partially supported, as food neophobia was associated with greater chemosensory impairment, whereas sensitivity to reward appeared to be associated with less risk of developing an alteration.

The worsening of alterations varied across domains, with the most frequent increases observed for discomfort (60.0%) and nausea (55.8%), followed by phantogeusia/parageusia (51.6%) and general taste alterations (45.3%). Lower worsening rates were observed for bitter/sour decline (32.6%), sweet/salty decline (26.3%), odour decline (24.2%), and hypergeusia (26.3%).

Sex and type of chemotherapy did not significantly affect changes in alterations, while older age was associated with higher odour decline.

This study provides new evidence of the impact of personality traits on general taste alterations, phantogeusia/parageusia, and nausea experienced after nine weeks of chemotherapy, suggesting that psychological factors may play a role in how patients perceive and cope with sensory changes during treatment.

To our knowledge, this is the first study reporting an association between higher sensitivity to reward and a lower likelihood of self-reported chemosensory alterations during chemotherapy. Specifically, patients with higher sensitivity to reward reported less severe general taste alterations, phantogeusia/parageusia, and nausea after nine weeks of treatment. One possible explanation is that individuals with a more active Behavioural Activation System preferentially orient their attention toward rewarding rather than aversive stimuli, thereby reducing the salience or perceived impact of treatment-related sensory symptoms. Indeed, sensitivity to reward reflects responsiveness to positive stimuli and approach motivation [44]. Consequently, individuals with higher sensitivity to reward may maintain the rewarding value of food and remain more engaged in eating despite unpleasant sensory changes [37, 45]. However, because chemosensory alterations were assessed using a self-report questionnaire, it cannot be determined whether this association reflects true differences in sensory dysfunction or differences in symptom perception, appraisal, and reporting.

Patients with higher levels of food neophobia were more likely to report general taste alterations, suggesting that individuals who are typically more reluctant toward unfamiliar foods may also be more sensitive to changes in their sensory experience during chemotherapy. This aligns with literature linking neophobia to heightened vigilance and reduced affective responses to food stimuli [24, 26–28, 30, 36, 45]. A noteworthy finding concerns the differential role of food neophobia across analytical approaches. While it did not predict the severity of sensory alterations, it consistently predicted their likelihood. This suggests that food neophobia may primarily influence the likelihood of perceiving and reporting sensory changes rather than their perceived severity. From a theoretical perspective, this interpretation is consistent with the characterization of food neophobia as a trait associated with avoidance, heightened vigilance, and increased sensitivity to potentially aversive stimuli. In this sense, neophobia may influence the cognitive–affective appraisal and reporting of chemosensory symptoms rather than the underlying sensory dysfunction itself. Similar mechanisms have been described in the broader literature on symptom perception, showing that psychological traits can shape the interpretation and reporting of bodily sensations independently of their objective physiological severity [46].

The identification of associations between personality traits and self-reported chemosensory alterations has potentially relevant clinical implications. Chemotherapy-induced chemosensory alterations are associated with reduced quality of life, impaired food intake, and an increased risk of malnutrition [16, 18]. Identifying patients who may be more vulnerable to these alterations could support the implementation of more personalized nutritional counselling and supportive care strategies.

From a clinical perspective, the assessment of personality traits before chemotherapy may help identify patients who are at greater risk of experiencing treatment-related chemosensory alterations. For example, patients with high food neophobia may benefit from early nutritional counselling aimed at preserving dietary variety by emphasizing familiar and acceptable foods, gradually introducing suitable alternatives when preferred foods become unpalatable, and providing practical strategies to maintain adequate nutritional intake. Conversely, patients with higher sensitivity to reward may require less intensive nutritional support, as they appear to perceive or report a lower burden of treatment-related chemosensory alterations.

Although these potential applications require prospective validation, our findings suggest that considering food-related personality traits alongside established clinical characteristics may contribute to a more individualized approach to supportive cancer care. At present, these findings should not be interpreted as supporting routine personality screening in oncology practice. Rather, they provide a rationale for future intervention studies evaluating whether tailoring nutritional counselling according to individual personality traits improves dietary adherence, food enjoyment, and nutritional outcomes during chemotherapy. The exact mechanisms by which personality traits such as food neophobia and sensitivity to reward may contribute to the reporting of chemosensory alterations during cancer treatment remain unclear. In addition to sensory changes, cancer treatment may modify how patients experience food, making it less enjoyable or even aversive. These changes may interact with personality traits and influence how sensory alterations are perceived and reported. As a result, the observed associations may reflect both biological effects of chemotherapy and changes in patients’ experience of eating. Further research is needed on larger samples to explore and clarify these associations and better understand the mechanisms.

This study also adds to the literature on demographic factors, suggesting a limited role of age and sex in sensory alterations. Older participants were more likely to report odour decline and general taste alterations but not other types of alterations. However, although age was included as a covariate in all analyses, the relatively wide age range of the sample may have masked age-related differences in personality expression, coping strategies, and emotional adaptation. Future studies with larger samples should examine whether the associations between personality traits and chemotherapy-induced chemosensory alterations differ across age groups. Chemotherapy regimen showed limited effects overall, with a significant association observed only for nausea in anthracycline-based and targeted therapies, although these findings should be interpreted with caution due to the use of combined treatment regimens and to the unbalanced sample.

While the study has important strengths in exploring the role of personality traits associated with chemosensory alterations for the first time, it presents also some limitations. First, similarly to many previous studies, the sample is unbalanced by gender, cancer type and chemotherapy regimen due to difficulties in recruitment of these vulnerable populations. The relatively small sample size and the predominance of female participants (86%) limit the generalizability of our findings, particularly to male patients. Future studies including larger and more sex-balanced cohorts are needed to confirm the present results. Secondly, other psychological factors (e.g., other personality traits) not assessed in the present study may also contribute to the variability in the changes in sensory alterations, as well as other demographic and clinical variables such as cancer stage. Indeed, eating is not only affected by sensory changes but also by emotional and contextual factors, often involving reduced pleasure, aversion, or altered meaning of food [47]. Disease stage may represent a relevant confounding factor, as it is often associated with differences in treatment intensity, type of chemotherapy regimens, overall health status, and symptom burden, all of which could influence the presence and severity of chemosensory alterations. These factors may influence both the perception and reporting of chemosensory alterations, so that further studies might address the role of additional factors on self-reported sensory alterations. Furthermore, although measures of food-related variables were collected, the relatively short period of 9 weeks may have limited the ability to detect changes in dietary intake and nutritional status. In addition, the evolving relationship with food during cancer and its treatment is complex and may require longer-term assessment to fully capture how traits such as food neophobia influence eating behaviour and nutritional outcomes. Future research should incorporate qualitative methods to further validate the statistical findings reported in this study.

## Conclusions

This study showed a significant increase in self-reported chemosensory alterations after nine weeks of chemotherapy and provides evidence that personality traits contribute to individual differences in both the likelihood and severity of these alterations. Food neophobia was associated with increased reporting, while sensitivity to reward appeared to be associated with minor reported alterations. These findings support a more personalized approach to supportive cancer care, where psychological traits are considered alongside clinical factors to better identify patients at risk of adverse sensory experiences and related nutritional complications. This could include tailored nutritional counselling, strategies to reduce food avoidance and aversive responses, and interventions aimed at preserving food enjoyment and motivation to eat according to patients' individual food-related traits.

## Supplementary Information

Below is the link to the electronic supplementary material.

Supplementary file1 (DOCX 137 KB)

## Data Availability

The data that support the findings of this study are available on request from the corresponding author, AL.
