# Supplementary material for: Personality traits are associated with the development and severity of chemosensory alterations during chemotherapy: a longitudinal study
Source: Support Care Cancer. 2026 Aug 10;34(9):838. doi: 10.1007/s00520-026-11074-2 (PMC13457425; doi:10.1007/s00520-026-11074-2)
Supplement: Supplementary file 1 [file 520_2026_11074_MOESM1_ESM.docx]

**Supplementary Material**

1. **Factor Analysis**

**CITAS Factor Analysis Results**

**Exploratory factor analysis**

An exploratory factor analysis (EFA) was conducted to investigate the latent structure of the scale. Analyses were performed using maximum likelihood extraction on the Pearson correlation matrix.

Data suitability for factor analysis was confirmed (KMO = 0.844; Bartlett’s test of sphericity: χ²(276) = 1825.88, p < 0.001), indicating adequate sampling and factorability of the correlation matrix.

**Factor retention**

The number of factors to retain was determined based on multiple criteria:

Eigenvalues > 1 (Kaiser criterion)

Scree plot inspection

The initial solution suggested four factors with eigenvalues greater than 1 (9.16, 3.39, 1.42, and 1.23). A fifth factor had an eigenvalue close to 1 (0.92), indicating a potential additional dimension.

The first four factors explained:

Factor 1: 38.18% of variance

Factor 2: 14.11%

Factor 3: 5.92%

Factor 4: 5.12%

The scree plot showed a clear inflection after the first factor, followed by a gradual decline consistent with a multidimensional structure. Although only four factors met the strict Kaiser criterion, a five-factor solution was retained based on interpretability and theoretical coherence. The five factors together accounted for 67.15% of the total variance.

**Factor rotation and interpretation**

An oblique rotation (Promax) was applied, as the factors were expected to be correlated due to conceptual overlap between sensory perception domains. Items were assigned to factors using a loading threshold of 0.32, following recommendations by Comrey and Lee (2013).

**Factor structure**

The rotated solution revealed a clear and interpretable five-factor structure:

Factor 1 – General gustatory dysfunction

(e.g., difficulty tasting food, altered taste perception)

Factor 2 – Olfactory dysfunction

(e.g., difficulty detecting general, food, floral, and body odours)

Factor 3 – Basic taste perception (sour/bitter)

(specific deficits in perceiving acidity and bitterness)

Factor 4 – Eating-related discomfort

(e.g., difficulty eating certain foods, reduced appetite, food-related discomfort)

Factor 5 – Dysgeusia and abnormal taste sensations

(e.g., bitter or metallic taste, persistent unpleasant taste, nausea-related sensations)

Table 1s. Items and factor pattern of the CiTAS scale. * Indicates an item added compared to the original version.

Most items loaded strongly on a single factor, supporting a well-defined multidimensional structure.

**Problematic items and model refinement**

Overall, the factor solution was satisfactory, with most items showing strong and interpretable loadings. A small number of items showed moderate cross-loadings or weaker specificity (e.g., items related to general taste alterations), but all items exceeded the minimum loading threshold on at least one factor with the exception of hypergeusia. However, given their conceptual relevance, no items were removed, and the full scale was retained.

Items loaded on five factors: (1) general taste alterations (GTA) and sweet/salty decline; (2) odour items; (3) bitter/sour decline, including weak loading of the hypergeusia item; (4) discomfort items (excluding nausea); and (5) nausea together with phantogeusia/parageusia. Based on these results, nausea was analyzed separately from discomfort, and hypergeusia and bitter/sour decline were treated as distinct constructs, so as sweet/salty decline and general taste alterations.

**Internal consistency**

Internal consistency was assessed using Cronbach’s alpha:

Factor 1: α = 0.892

Factor 2: α = 0.907

Factor 3: α = 0.799

Factor 4: α = 0.805

Factor 5: α = 0.907

These values indicate good to excellent internal consistency across all factors.

2. **Table reporting mean, SD, and correlations at T0 and T1 for each CiTAS subscale**

|  | **T0 mean (SD)** | **T1 mean (SD)** | **r (T0, T1)** | **p** |
| --- | --- | --- | --- | --- |
| Sweet/Salty Decline | 1.068 (0.331) | 1.421 (0.780) | 0.186 | 0.071 |
| Bitter/Sour Decline | 1.174 (0.600) | 1.495 (0.843) | 0.139 | 0.181 |
| Odour Decline | 1.206 (0.621) | 1.352 (0.730) | **0.479** | **<0.0001** |
| General Taste Alterations | 1.208 (0.468) | 1.818 (1.077) | **0.223** | **0.030** |
| Phanto/Parageusia | 1.226 (0.591) | 1.982 (1.269) | **0.229** | **0.026** |
| Nausea | 1.263 (0.802) | 2.263 (1.354) | -0.045 | 0.666 |
| Discomfort | 1.465 (0.691) | 1.989 (1.011) | **0.457** | **<0.0001** |
| Hypergeusia | 1.305 (0.864) | 1.747 (1.280) | 0.196 | 0.057 |
